# Supplementary material for: Second-generation lung-on-a-chip with an array of stretchable alveoli made with a biological membrane
Source: Commun Biol. 2021 Feb 5;4:168. doi: 10.1038/s42003-021-01695-0 (PMC7864995; doi:10.1038/s42003-021-01695-0)
Supplement: Supplementary file 2 — Supplementary Information [file 42003_2021_1695_MOESM2_ESM.pdf]

Supplementary information

Second-generation lung-on-chip with an array of stretchable alveoli made with a biological membrane

Authors: Pauline Zamprogno[1], Simon Wüthrich[1], Sven Achenbach[1], Giuditta Thoma[1], Janick D. Stucki[1,6], Nina Hobi[1,6], Nicole Schneider-Daum[2], Claus-Michael Lehr[2], Hanno Huwer[3], Thomas Geiser[4], Ralph A. Schmid[5], Olivier T. Guenat[1,4,5]*

Affiliations:
[1] Organs-on-Chip Technologies Laboratory, ARTORG Center, University of Bern, Murtenstrasse 50, 3008 Bern, Switzerland
[2] Drug Delivery (DDEL), Helmholtz-Institute for Pharmaceutical Research Saarland (HIPS), Campus E8 1, 66123 Saarbrücken, Germany
[3] SHG Clinics, Department of Cardiothoracic Surgery, Völklingen Heart Center, Pasteurstrasse 11, 66333 Völklingen, Germany
[4] Department of Pulmonary Medicine, University Hospital of Bern, Murtenstrasse 35, 3008 Bern, Switzerland
[5] Department of General Thoracic Surgery, University Hospital of Bern, Murtenstrasse 35, 3008 Bern, Switzerland
[6] AlveoliX AG, Freiburgstrasse 3, 3010 Bern, Switzerland

*E-mail: olivier.guenat@artorg.unibe.ch

Supplementary Figure 1: Size of the collagen-elastin fibers
The collagen-elastin membrane structure was investigated by SEM. A minimum of four pictures were taken per membranes. The number of fibers were identified according to their size with ImageJ. The average fiber size is 45.7 ± 18.9 nm (n=2).

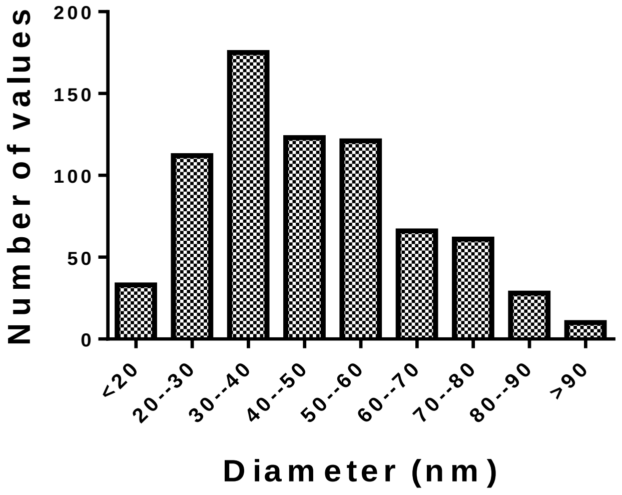

Supplementary Figure 2: Chip design (made on Solidworks)

The device was made of a top PDMS layer (apical chamber), a collagen-elastin membrane supported by a gold mesh and a polycarbonate bottom (basolateral chamber). The alignment of the layer was ensured by two dowels pins.

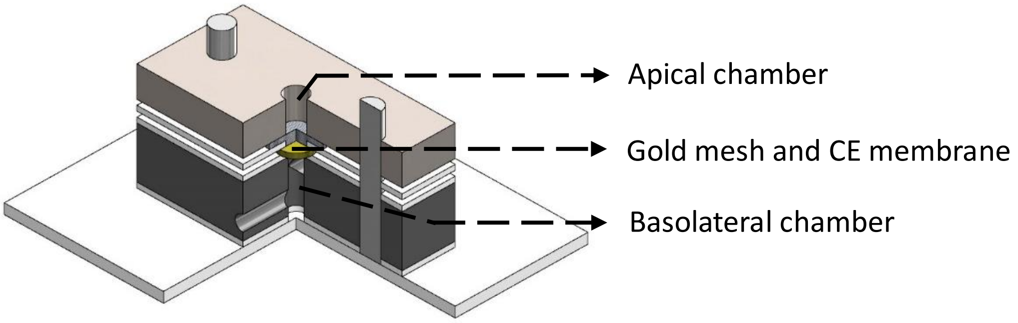

Supplementary Figure 3: Thickness of the CE-membrane in function of its composition

The thickness of the CE-membranes made of different ratio of collagen and elastin was evaluated by reflective light. The CE-membrane with a 1:1 (resp. a 2:1) ratio had a final concentration of collagen and of elastin of 3.5 $mg.mL^{-1}$ each (resp. 3.5 $mg.mL^{-1}$ of collagen and 1.75 $mg.mL^{-1}$ of elastin). The collagen membrane was only made of collagen type I with a final concentration of 3.5 $mg.mL^{-1}$. A volume of 1.6 $\mu l.mm^{-2}$ was used for each condition. The CE-membrane with a 1:1 ratio had a thickness of 7.7 ± 1.7 µm, while that with a 2:1 ratio was 4.9 ± 0.6 µm thin. The collagen membrane was 3.2 ± 0.5 µm thin.

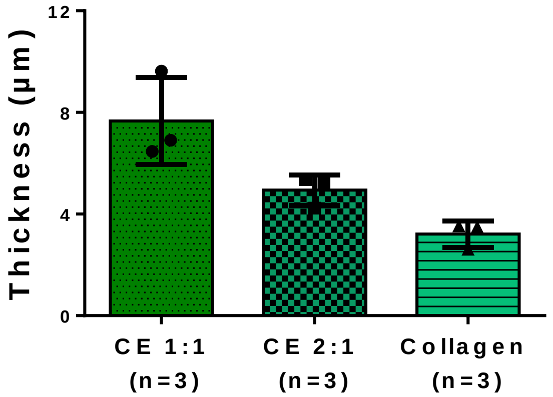

Supplementary Figure 4: Reproducibility of the CE membranes thicknesses (ratio 1:1, 1.6 µL.mm^{-2})

Several membranes made of collagen and elastin (ratio 1:1) were produced by pipetting 1.6 µL.mm^{-2} of solution on top of the gold mesh. After drying, their respective thicknesses were evaluated. Four to six measurements were taken in several hexagons across the mesh. The thickness variation across the hexagon array was below 20%. This variation is mainly due to the location of the measurement, as the membrane is thinner in the centre of the hexagon.

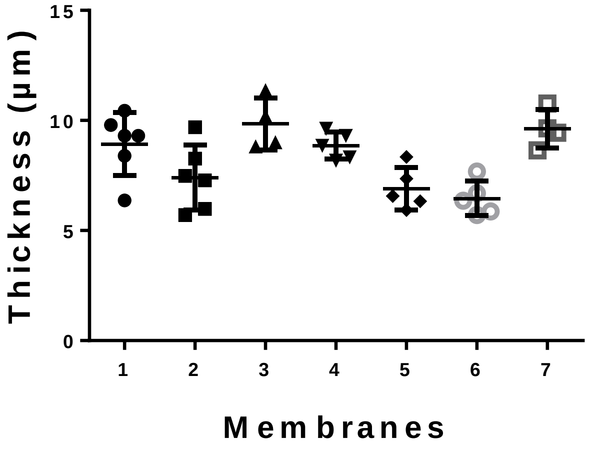

Supplementary Figure 5: Spectral absorbance of the CE- and of a polyester membrane

The spectral absorbance of a CE- membrane (10µm-thin with a 1:1 and a 2:1 ratio) and a collagen membrane was smaller than that of a polyester membrane used in standard cell culture inserts.

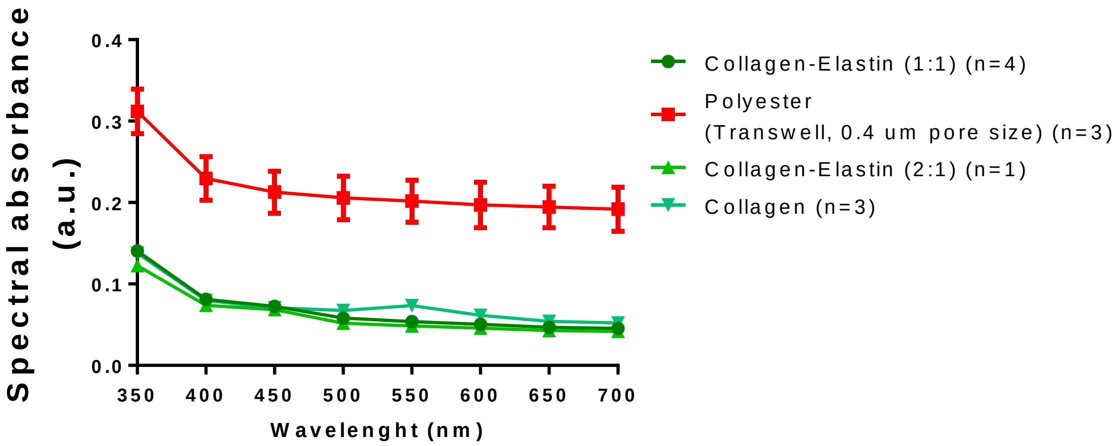

Supplementary Figure 6: Absorption of Rhodamine B in membranes made of biological and polymeric material

We evaluated the absorption of Rhodamine B (10 µM in PBS) in membranes made of several polymeric and biological materials. The membranes were incubated during 2h in presence of Rhodamine B. It results that the tested polymers (PDMS and polyester) absorbed more Rhodamine B than the tested biological materials. The absorption also varied in function of the thickness of the material and on its porosity. A 40 µm-thin PDMS membrane absorbed 2.4 more RhoB molecules than a 10 µm-thin PDMS membrane. A Polyester membrane with 3µm pores and a pore density of 2 x 10^6 pores.cm^{-2}, absorbed 2.5 more RhoB molecules than the same membrane with 0.4 µm pores and a pore density of 4 x 10^6 pores.cm^{-2} according to the supplier (https://betastatic.fishersci.com/content/dam/fishersci/en_US/documents/programs/scientific/brochures-and-catalogs/guides/corning-transwell-permeable-supports-guide.pdf).

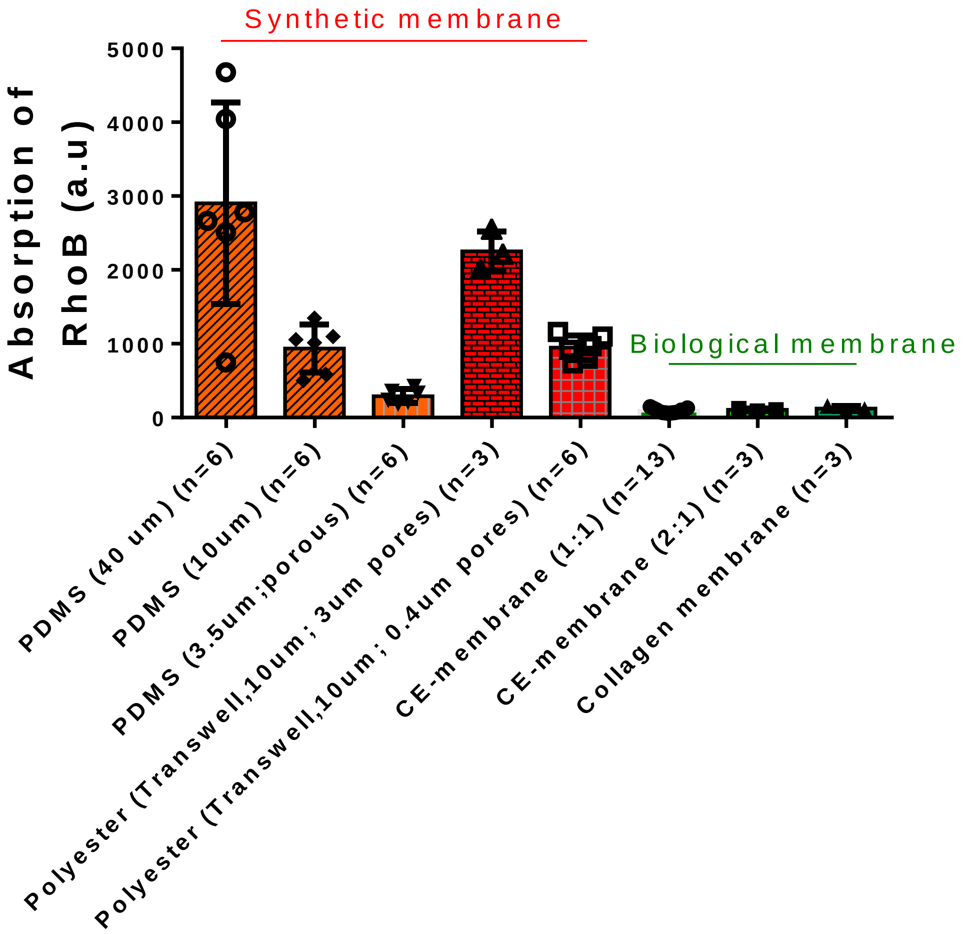

Supplementary Figure 7: Three-dimensional deflection of the CE-membrane with a monolayer of endothelial cells

A monolayer of RFP-labelled human lung microvascular endothelial cells, cultured on the CE-membrane (ratio 1:1), was imaged at rest position (no mechanical stress, top view) and stretched with a negative pressure of 2 kPa (bottom view).

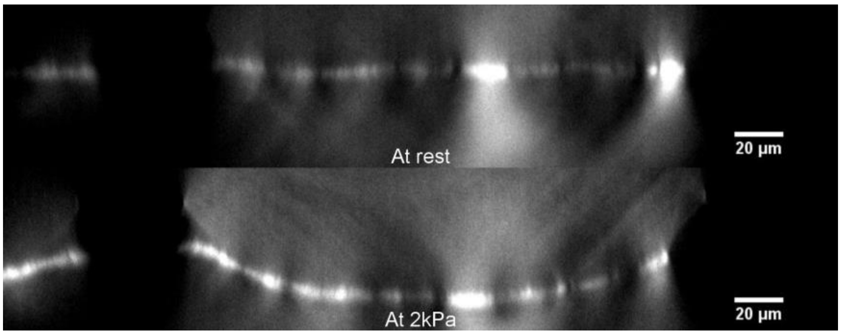

Supplementary Figure 8: Maximum linear strain without (control) and with confluent lung alveolar epithelial cells of two patients at two pressures.

The hAEpC form a confluent tight layer which influence the stretchability of the membrane. At -2 kPa, without cell, the linear strain was 10.7% ± 1.1% while it only reached 3.2% ± 0.9% with hAEpC. By increasing the pressure at -4 kPa the linear strain reached 9.9% ± 1.6%.

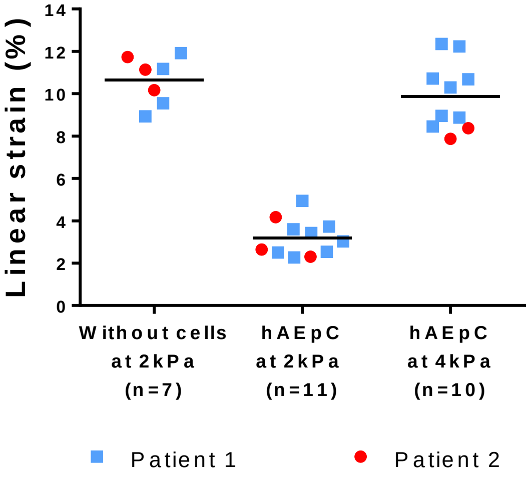

Supplementary Figure 9: Maximum linear strain in function of the applied vacuum and of the CE-membrane composition.

CE-membranes of three different compositions were tested with a vacuum of -1, -1.5 and -2kPa, compared to a 10um thin PDMS membrane. At equal pressure, the CE-membrane with higher collagen concentration deflected less than the CE-membranes with lower collagen concentration. For example, at -1.5kPa, the radial strain was 5.0% ± 1.4% for a 2:1 ratio, it reached 7.6% ± 1.2% for a 1:1 ratio and 13.1% ± 1.9% for a ratio 1:3.

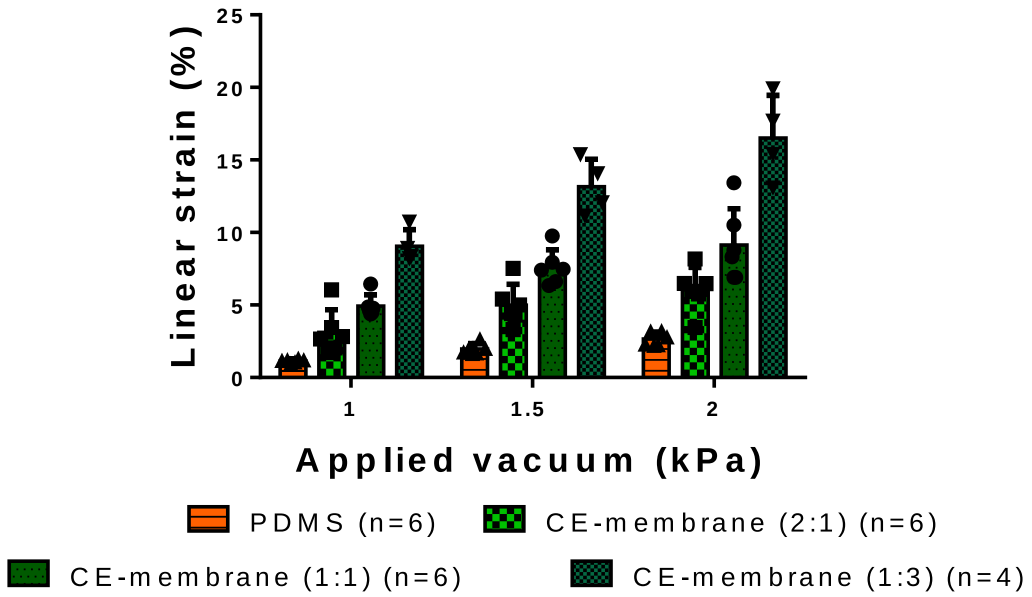

Supplementary Figure 10: Deflection of CE-membrane exposed to MMP-8 in function of time.

CE-membranes have been exposed to MMP-8 at 10 $U.mL^{-1}$ during 45 min and 1h, and tested with a vacuum at -1kPa. Membranes without treatment have been used as control. After 1h of treatment, the membrane deflected at 46.7 ± 1.7µm, whereas the deflection reached only 35.6 ± 3.6µm after 45 min and 31.0 ± 2.3µm without MMP-8.

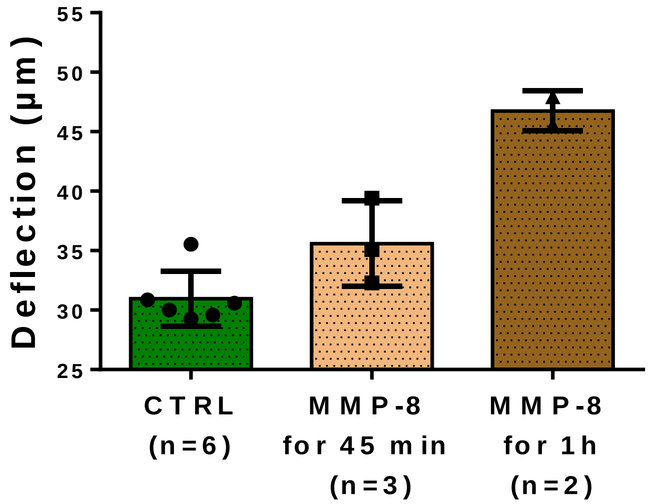

Supplementary Figure 11: Viability of hAEpC at day 8 after 4 days at ALI

hAEpC were cultured on the CE membrane in submerged condition (ILI) or Air liquid condition (ALI). In ILI condition, cells were cultured with physiological medium on the apical and the basal chambers for 8 days. In ALI condition, cells were submerged for 4 days, then the medium was removed from the apical side and replaced in the basolateral chamber. The cells were then cultured for 4 days at the air-liquid interface, with the nutrients being provided by diffusion from the basal chamber through the membrane. Culturing the hAEpC at ALI had no impact on the viability of the cells or on the barrier formation, as shown below by the expression of tight junctions (ZO-1).

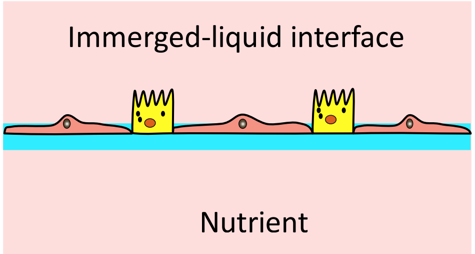

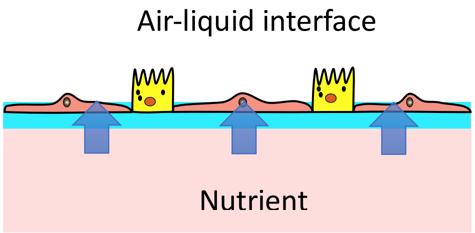

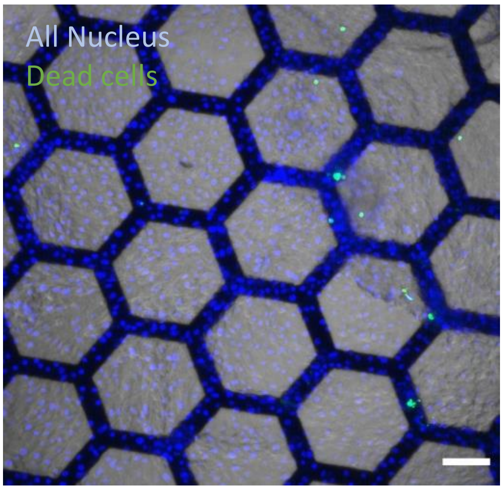

hAEpC at day 8 in ILI condition; nuclei (blue) and dead cells (green)

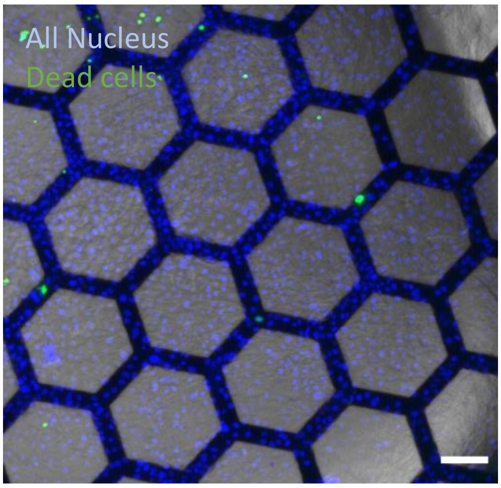

hAEpC at day 8 in ALI condition; nuclei (blue) and dead cells (green)

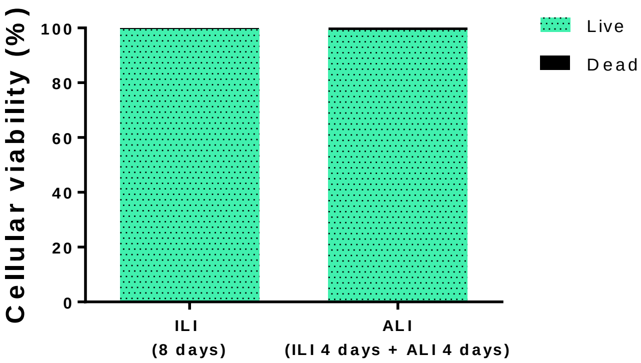

Viability of hAEpC in monoculture at day 8 in ILI and ALI condition. ILI: n=2 (with 633 and 567 cells measured respectively) and ALI: n=2 (with 675 and 689 cells measured respectively).

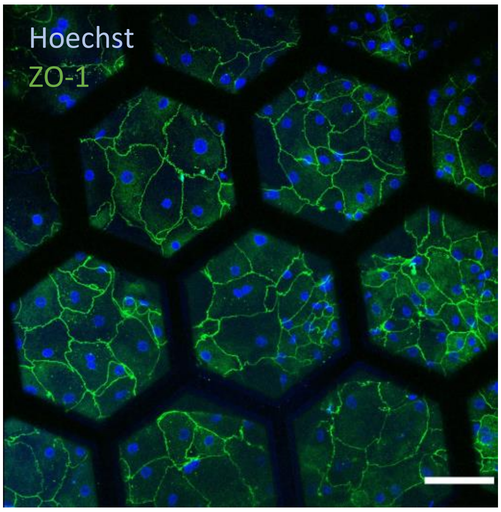

hAEpC at day 8 in ALI condition; nuclei (Hoechst; blue) and tight junction (ZO-1; green). Scale bar: 100µm

Supplementary Figure 12: TEM imaging of hAEpC at different time points

Human primary alveolar cells (hAEpC) were successfully cultured on the CE-membrane. The cells adherence was excellent on the membrane and did not detach even after one week in culture. Lamellar-like bodies can be seen from day 0 (D0) to day 8 (D8).

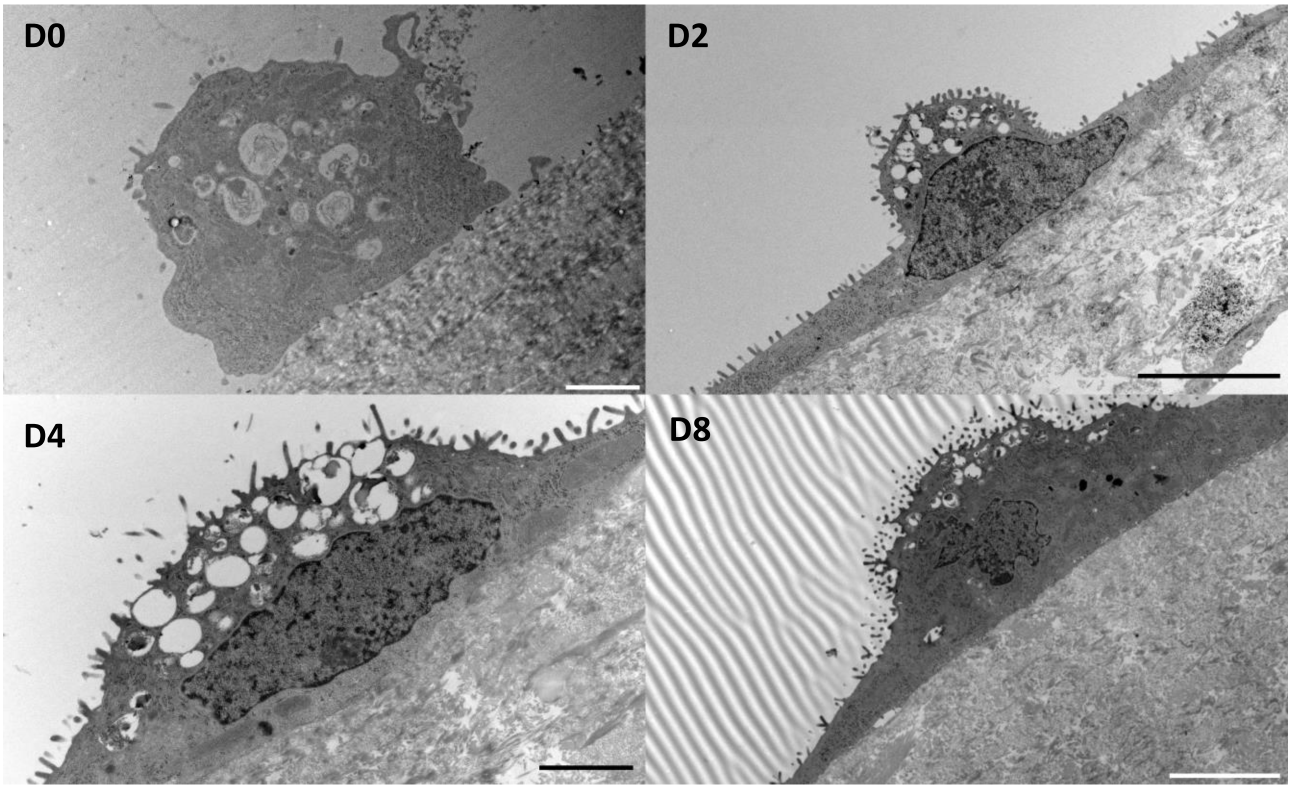

hAEpC at Day 0. Scale bar: 2µm; Day 2. Scale bar: 5µm; Day 4. Scale bar: 2µm; Day 8. Scale bar: 5µm

Supplementary Figure 13: **Attachment of VeraVec cells on CE-membrane**

The primary lung endothelial cells were cultured on the CE-membrane and PDMS membrane for 8 days. Their adherence and the interaction with the support was checked by the activation of focal adhesion kinase (FAK). The autophosphorylation of Tyrosine 397, a site involve in the activation of FAK, was visualized by immunofluorescence. No significant difference was observed between the results obtained with the collagen 1 - fibronectin coated PDMS and the CE-membrane. This may be due to the similarity of the composition of the coating and of the CE-membrane.

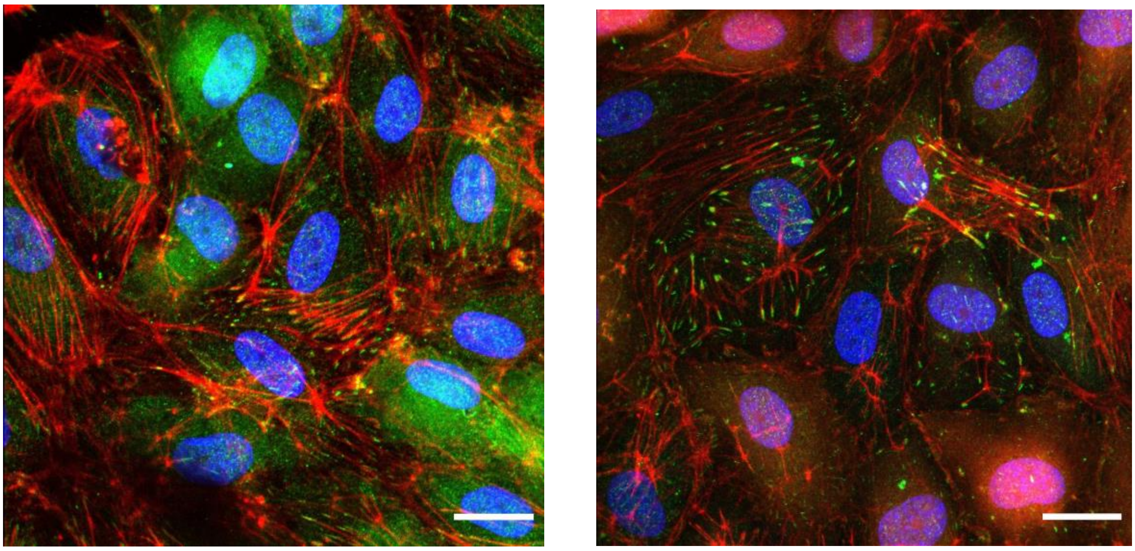

VeraVec cells at day 8 on CE-membrane (left) and on PDMS (right) with Tyr397 (green), Hoechst (Blue), Actin (red). Scale bar: 20 µm

Supplementary Figure 14: Surface and number of hAEpC in function of the seeding concentration

The hAEpC were seeded at high concentration (HC, 270'000 cells.cm^{-2}) and low concentration (LC, 100'000 cells.cm^{-2}). The cellular surface was quantified using ZO-1 (green) to show the cell borders and Hoechst (blue) for the nuclei. When cells were seeded at high concentration, they almost reached confluence at day 2 and their number remains stable (day 8). However, when they were seeded at LC, their number increased significantly between day 2 and day 8.

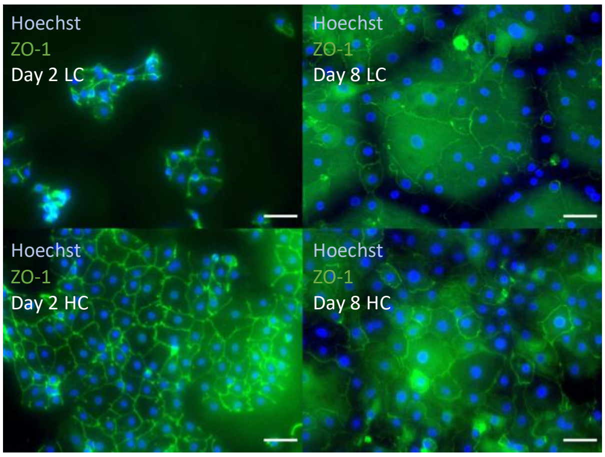

Day 2 LC (top left); day 8 LC (top right); Day 2 HC (bottom left); Day 8 HC (bottom right). Scale bar: 50 µm

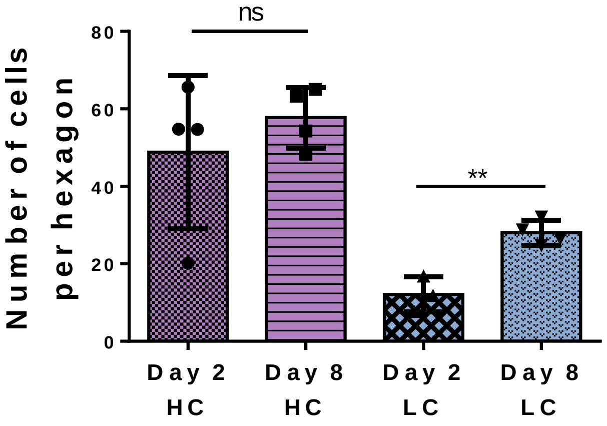

Number of hAEpC per hexagon at day 2 and 8 in function of the cell seeding concentration. Day 2, HC: n=4 (with 7, 5, 7 and 3 hexagons measured respectively); Day 8, HC: n=4 (with 6, 7, 11 and 6 hexagons measured respectively); Day 2, LC: n=3 (with 7, 10 and 12 hexagons measured respectively) and Day 8, LC: n=4 (with 8, 9, 9 and 9 hexagons measured respectively).

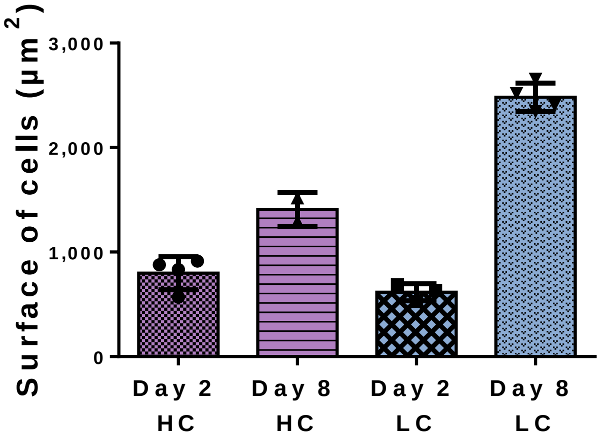

Cellular surface of hAEpC at day 2 and 8 in function of the cell seeding concentration. Day 2, HC: n=4 with 188 cells measured (32, 67, 45 and 44 respectively); Day 8, HC: n=2 with 86 cells measured (25 and 61 respectively); Day 2, LC: n=3 with 86 cells measured (17, 33 and 36 respectively) and Day 8, LC: n=4 with 188 cells measured (62, 37, 39 and 50 respectively).

Supplementary Figure 15: Long-term cultures of hAEpC and of primary endothelial cells
hAEpC and primary lung endothelial cells were cultured on the CE-membrane for up to 3 weeks without any impact on their viability. At day 21, hAEpC cells still clearly expressed tight junction marker (ZO-1) showing the maintenance of a tight barrier.

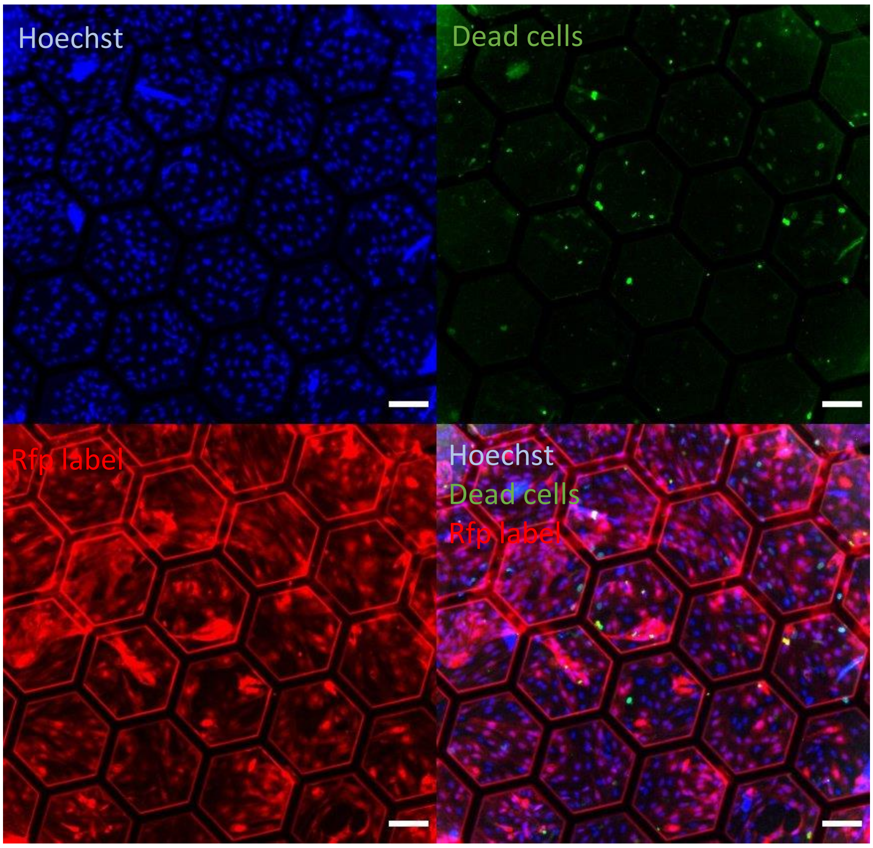

VeraVec cells at day 21. All nuclei (top left); Dead cells (top right); RFP-label endothelial cells (bottom left); Merged picture (bottom right). Scale bar: 100µm

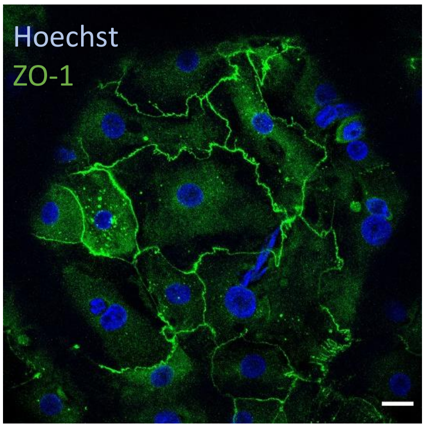

hAEpC at day 21. ZO-1 (green), Hoechst (Blue). Scale bar: 20µm

Supplementary Figure 16: ZO-1 expression on 4 patients' cells

The formation of tight junction, via the expression of ZO-1, was tested with cells from four patients. A control experiment was carried out on PDMS with the same cells with similar results.

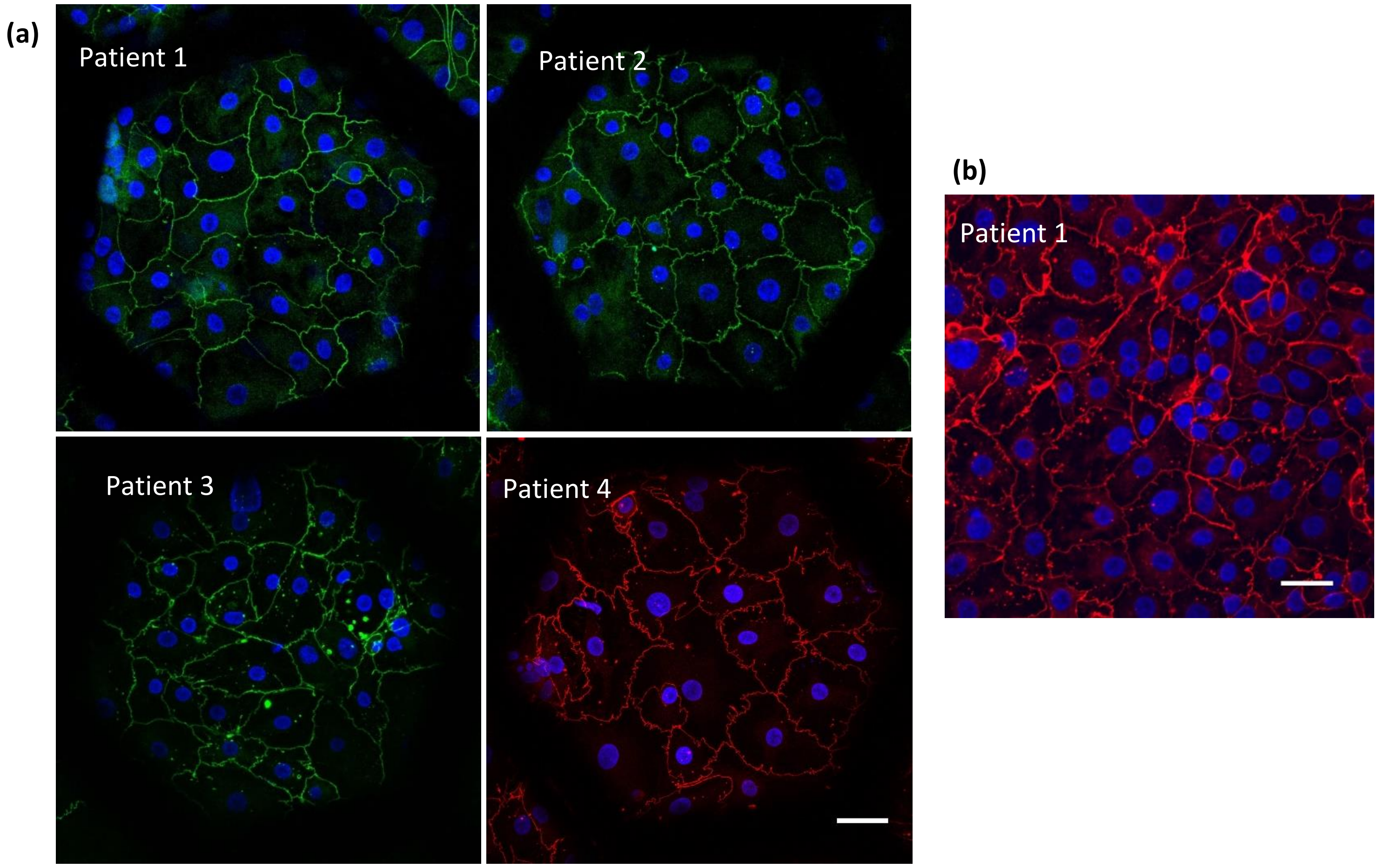

Sup. Fig. 16: Tight junctions (ZO-1) expressed in cells from five patients: (a) cells from four patients were cultured on the CE-membrane with nuclei in blue and ZO-1 in green for patients 1-3 and in red for patient 4, (b) cells from patient 1 were cultured on a PDMS membrane with nucleus and ZO-1 in red. Scale bar: 30µm

Supplementary Figure 17: Calculation of the linear mechanical stress

A simple mathematical model was used to evaluate the mechanical stress of the cells cultured on the membrane. The deflection of the membrane was approximate as a circular segment. On each hexagon, the absolute deflection of the membrane (h) is given by the difference between the deflection of the membrane (1) and the gold mesh (2) (Eq. 1). The length of the stretched membrane (L) can be approximated with a circular segment and it was calculated with the formula (Eq. 2) with (L0) the original length. The change in length (ΔL) can be calculated with the formula (Eq. 3). Finally, the linear strain (ε) is the ratio of change in length to the origin length (Eq. 4).

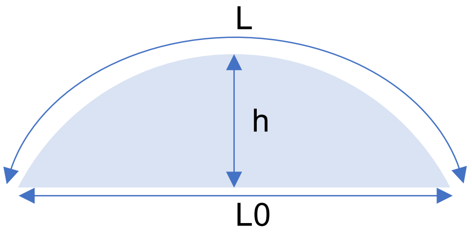

$$Equation\ 1: \quad h = h1 - h2$$

$$Equation\ 2: \quad L = \frac{\arctan\left(\frac{2*h}{L0}\right) * (4*h^2 + L0^2)}{2*h}$$

$$Equation\ 3: \quad \Delta L = L - L0$$

$$Equation\ 4: \quad \varepsilon\ [\%] = \frac{\Delta L}{L0} * 100$$

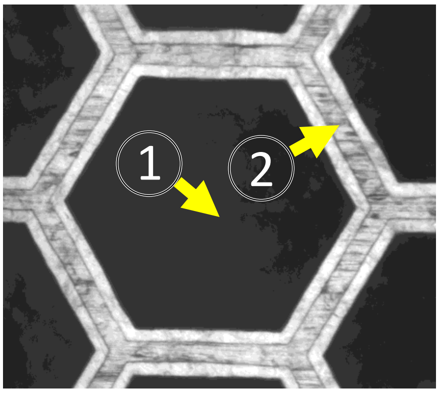

Measurements points (1: deflection of the CE-membrane; 2: deflection of the gold mesh)
