# Supplementary material for: Second-generation lung-on-a-chip with an array of stretchable alveoli made with a biological membrane
Source: Commun Biol. 2021 Feb 5;4:168. doi: 10.1038/s42003-021-01695-0 (PMC7864995; doi:10.1038/s42003-021-01695-0)
Supplement: Supplementary file 3 — Description of Supplementary Files [file 42003_2021_1695_MOESM3_ESM.pdf]

Description of Additional Supplementary Files

File name: Supplementary Movie 1

Description: 3D deflection of human primary cells on the CE-membrane.

File name: Supplementary Data 1

Description: The source data underlying figures.
