# Supplementary material for: Second-generation lung-on-a-chip with an array of stretchable alveoli made with a biological membrane
Source: Commun Biol. 2021 Feb 5;4:168. doi: 10.1038/s42003-021-01695-0 (PMC7864995; doi:10.1038/s42003-021-01695-0)
Supplement: Supplementary file 6 — Reporting Summary [file 42003_2021_1695_MOESM6_ESM.pdf]

nature research

Corresponding author(s): Prof. Dr. Olivier Guenat

Last updated by author(s): 2020.12.15

Reporting Summary

Nature Research wishes to improve the reproducibility of the work that we publish. This form provides structure for consistency and transparency in reporting. For further information on Nature Research policies, see Authors & Referees and the Editorial Policy Checklist .

Statistics

For all statistical analyses, confirm that the following items are present in the figure legend, table legend, main text, or Methods section.

n/a	Confirmed	
☐	☒	The exact sample size (*n*) for each experimental group/condition, given as a discrete number and unit of measurement
☐	☒	A statement on whether measurements were taken from distinct samples or whether the same sample was measured repeatedly
☐	☒	The statistical test(s) used AND whether they are one- or two-sided<br>*Only common tests should be described solely by name; describe more complex techniques in the Methods section.*
☐	☒	A description of all covariates tested
☒	☐	A description of any assumptions or corrections, such as tests of normality and adjustment for multiple comparisons
☐	☒	A full description of the statistical parameters including central tendency (e.g. means) or other basic estimates (e.g. regression coefficient) AND variation (e.g. standard deviation) or associated estimates of uncertainty (e.g. confidence intervals)
☒	☐	For null hypothesis testing, the test statistic (e.g. *F*, *t*, *r*) with confidence intervals, effect sizes, degrees of freedom and *P* value noted<br>*Give P values as exact values whenever suitable.*
☒	☐	For Bayesian analysis, information on the choice of priors and Markov chain Monte Carlo settings
☒	☐	For hierarchical and complex designs, identification of the appropriate level for tests and full reporting of outcomes
☒	☐	Estimates of effect sizes (e.g. Cohen's *d*, Pearson's *r*), indicating how they were calculated

Our web collection on statistics for biologists contains articles on many of the points above.

Software and code

Policy information about availability of computer code

Data collection	The following software have been used:<br>- Axiovision Rel. 4.8: measurement of the thickness and the quantification of the membrane deflection<br>- Tecan i-control software: evaluation of the fluorescence intensity (permeability assay, Absorption/adsorption assay) and the transparency of the membrane<br>- ZEN software: acquisition of all confocal pictures<br>- COMSOL Multiphysics 5.3: illustration of the membrane deflection
Data analysis	- Statistical analysis was performed using GraphPad Prism 6 software.<br>- ImageJ was used to evaluate the cells surface, number of cells (live and dead).<br>- Axiovision Rel. 4.8 was employed to evaluate the thickness of membrane

For manuscripts utilizing custom algorithms or software that are central to the research but not yet described in published literature, software must be made available to editors/reviewers. We strongly encourage code deposition in a community repository (e.g. GitHub). See the Nature Research guidelines for submitting code & software for further information.

Data

Policy information about availability of data

All manuscripts must include a data availability statement. This statement should provide the following information, where applicable:

- Accession codes, unique identifiers, or web links for publicly available datasets
- A list of figures that have associated raw data
- A description of any restrictions on data availability

The data acquired during this study are available in the file called "Source Data".

Some information has been judged unnecessary for the manuscript understanding, for example the mean and standard deviation for each data. However, this information can be provided by the corresponding author.

Field-specific reporting

Please select the one below that is the best fit for your research. If you are not sure, read the appropriate sections before making your selection.

- [x] Life sciences
- [ ] Behavioural & social sciences
- [ ] Ecological, evolutionary & environmental sciences

For a reference copy of the document with all sections, see nature.com/documents/nr-reporting-summary-flat.pdf

Life sciences study design

All studies must disclose on these points even when the disclosure is negative.

Sample size	The sample size was chosen in function of the experiment. Typically, for the characterization of physical properties, such as the thickness or the deflection of the membrane, a low n number was sufficient to give a good representation of the parameter. In each case, the corresponding author can provide more information if required.
Data exclusions	No data was excluded.
Replication	The number of replicates depends on the experiment performed and is indicated in the legends and in the materials and methods section. Additional information can be provided by the corresponding author.
Randomization	All samples were randomly allocated into experimental groups.
Blinding	The blinding cannot be applied in this study. Typically, when a sample was randomly placed at a typical cell culture condition, such as air-liquid interface, the condition applied during the whole duration of the experiment.

Reporting for specific materials, systems and methods

We require information from authors about some types of materials, experimental systems and methods used in many studies. Here, indicate whether each material, system or method listed is relevant to your study. If you are not sure if a list item applies to your research, read the appropriate section before selecting a response.

Materials & experimental systems

n/a	Involved in the study	
[ ]	[x]	Antibodies
[ ]	[x]	Eukaryotic cell lines
[x]	[ ]	Palaeontology
[x]	[ ]	Animals and other organisms
[x]	[ ]	Human research participants
[x]	[ ]	Clinical data

Methods

n/a	Involved in the study	
[x]	[ ]	ChIP-seq
[x]	[ ]	Flow cytometry
[x]	[ ]	MRI-based neuroimaging

Antibodies

Antibodies used

During this study, the following antibodies have been used:
- ZO-1 Antibody; first antibody; Class: Monoclonal; Host: Mouse; Dilution: 1:100; Supplier: Invitrogen, Catalog number: 33-9100, Clone name: ZO1-1A12.
- SP-C Antibody, first antibody; Class: Polyclonal; Host: Rabbit; Dilution: 1:200; Supplier: Seven Hills Bioreagents; Catalog number: WRAB-76694.
- E-Cadherin; first antibody; Host:Rabbit ; Dilution: 1:200.
- KI-67; first antibody; Class: Monoclonal; Host: Mouse ; Dilution: 1:750; Supplier: Cell Signaling Technology ; Catalog number:9449.
- Phospho-FAK (Tyr397) Antibody; first antibody; Class: Polyclonal; Host: Rabbit; Dilution: 1:100; Supplier: Invitrogen; Catalog number: 44624G
- Acti-stain 488 phalloidin, Dilution: 1:150; Supplier: Cytoskeleton; Catalog number:PHDG1; Emission: 485nm/Exitation: 535nm.
- IgG-Alexa488 (Mouse), Secondary antibody; Class: Polyclonal; Host: Goat; Dilution: 1:500; Supplier: Invitrogen ; Catalog number: A-11001; Emission: 495nm/Exitation: 519nm.
- IgG-Alexa488 (Rabbit); Secondary antibody; Class: Polyclonal; Host: Goat; Dilution: 1:500; Supplier: Invitrogen ; Catalog number: A11008; Emission: 495nm/Exitation: 519nm.
- IgG-Alexa546 (Rabbit); Secondary antibody; Class: Polyclonal; Host: Goat; Dilution: 1:500; Supplier: Invitrogen ; Catalog number: A11035; Emission: 556nm/Exitation: 573nm.

- IgG-Alexa546 (Mouse); Secondary antibody; Class: Polyclonal; Host: Goat; Dilution: 1:500; Supplier: Invitrogen ; Catalog number:A11003; Emission: 556nm/Exitation: 573nm.
- Hoechst 33342; Dilution: 1:1000; Supplier: Invitrogen ; Catalog number: H3570; Emission: 350nm/Exitation: 461nm.

Validation

- ZO-1 Antibody: https://www.thermofisher.com/antibody/product/ZO-1-Antibody-clone-ZO1-1A12-Monoclonal/33-9100
- SP-C Antibody: https://www.sevenhillsbioreagents.com/products/anti-mature-sp-c-rabbit
- KI-67: https://www.cellsignal.com/products/primary-antibodies/ki-67-8d5-mouse-mab/9449
- Acti-stain 488 phalloidin: https://www.cytoskeleton.com/phdg1
- IgG-Alexa488 (Mouse): https://www.thermofisher.com/antibody/product/Goat-anti-Mouse-IgG-H-L-Cross-Adsorbed-Secondary-Antibody-Polyclonal/A-11001
- IgG-Alexa488 (Rabbit): https://www.thermofisher.com/antibody/product/Goat-anti-Rabbit-IgG-H-L-Cross-Adsorbed-Secondary-Antibody-Polyclonal/A-11008
- IgG-Alexa546 (Rabbit): https://www.thermofisher.com/antibody/product/Goat-anti-Rabbit-IgG-H-L-Highly-Cross-Adsorbed-Secondary-Antibody-Polyclonal/A-11035
- IgG-Alexa546 (Mouse): https://www.thermofisher.com/antibody/product/Goat-anti-Mouse-IgG-H-L-Cross-Adsorbed-Secondary-Antibody-Polyclonal/A-11003
- Hoechst 33342: https://www.thermofisher.com/order/catalog/product/H3570?SID=srch-srp-H3570

Eukaryotic cell lines

Policy information about cell lines

Cell line source(s)

Two human primary cell types have been used in this study:
- Human alveolar epithelial primary cells (hAEpC) isolated from tissue obtained from healthy areas removed from patients undergoing lung tumor resection surgery. All patients gave informed written consent for usage of surgical material for research purposes, which was approved by ethical committee from the Ärztekammer des Saarlandes. All procedures were carried out in accordance with institutional guidelines from Saarland (Germany) and from the Canton of Bern (Switzerland).
- RFP-labelled human lung microvascular endothelial cells (VeraVec)

Authentication

- hAEpC were characterized in the following paper: (Daum et al.; Human Cell Culture Protocols; 2012) and (Stucki et al.; Scientific Report; 2018).
- VeraVec were acquired from Angiocrine Biosciences Inc. (San Diego, CA, USA).

Mycoplasma contamination

The cells were not tested for mycoplasma contamination.

Commonly misidentified lines
(See ICLAC register)

Name any commonly misidentified cell lines used in the study and provide a rationale for their use.
